# Phylogenetic analysis of HDV isolates from HBsAg positive patients in Karachi, Pakistan

**DOI:** 10.1186/1743-422X-9-162

**Published:** 2012-08-15

**Authors:** Shadab Perveen, Muhammad Israr Nasir, Syed M Shahid, Abid Azhar, Obaid Yusuf Khan

**Affiliations:** 1 The Karachi Institute of Biotechnology & Genetic Engineering (KIBGE), University of Karachi, Karachi, Pakistan; 2 Dow University of Health Sciences (DUHS), Karachi, Pakistan; 3 Department of Genetics, University of Karachi, Karachi, Pakistan

**Keywords:** Hepatitis D virus, HDV- clades, Phylogeny, HDAg

## Abstract

**Background:**

In spite of a high occurrence of Hepatitis Delta in the province of Sindh in Pakistan, no genetic study of Hepatitis Delta virus (HDV) isolates from this region was carried out. The aim of this study is to analyze the genetic proximity within local HDV strains, and relationship with other clades of HDV, using phylogenetic analysis.

**Results:**

Phylogenetic analysis of nucleotide sequences of the Hepatitis Delta Antigen (HDAg) R0 region obtained in this study, showed considerable diversity among the local strains with a potential subgroup formation within clade I. The multiple sequence alignment of predicted amino acids within clade I showed many uncommon amino acid substitutions within some conserved regions that are crucial for replication and assembly of HDV.

**Conclusions:**

The studied strains showed a range of genetic diversity within HDV clade I. There is clustering of sequences into more than one group, along with formation of potential subgroup within clade I. Clustering shows the genetic closeness of strains and indicates a common origin of spread of HDV infection. Further phylogeny-based studies may provide more information about subgroup formation within clade I and may be used as an effective tool in checking and/or preventing the spread of hepatitis D virus infection in this region.

## Background

Hepatitis, the inflammation of liver can be caused by variety of viruses such as Hepatitis A, B, C, D and E viruses. Among these some are food borne, while some are transmitted only through contaminated blood and blood products [1].

Hepatitis D virus (HDV) or Delta was first identified as an antigen in liver biopsies of chronic hepatitis B infected patients. This antigen was named as Delta antigen that required mandatory existence of Hepatitis B virus (HBV). The source of this antigen was later identified as a RNA virus that was a satellite virus of HBV. Coexistence of HDV with HBV increases the rate of cirrhosis and hepatocellular carcinoma many folds as compared to hepatitis B alone. HDV has two modes of infection, co-infection or a super infection [2,3].

Hepatitis delta virus (HDV) is the smallest RNA satellite virus identified to date consisting of 1700 nucleotides [4,5]. The HDV RNA genome is divided into two regions; a viroid-like conserved region that is infectious and does not encode any protein while the other part of genome codes for the only known functional protein called the Hepatitis Delta Antigen (HDAg). HDAg is known to exist in two essential forms [4,5], the smaller S-HDAg (195 a.a; 24KDa) and the larger L-HDAg (214a.a; 27KDa). These two proteins are encoded by a single gene with the larger protein arising as a result of extension of the coding sequence due to RNA editing at the amber/W site of HDV antigenomic RNA [4,6,7].

The HDV RNA from nucleotide 900 to 1260 bp (corresponding to amino acids 111-214 of HDAg) encompasses several important functional elements and has been used to explore genetic diversity among Hepatitis D Virus [5,8]. This includes one of the two RNA binding domain comprising Arginine Rich Motif (ARM) extending from amino acid position138-145, and its facilitating region Helix loop helix motif (HLH) from 111-137 amino acids. The Glycine-proline rich region (coiled-coil motif), is a characteristic feature of transcriptional factors and facilitates greatly in formation of HDAg–RNA complex [8,9]. The amino acids at C-terminal position of S-HDAg carry the epitope region which has a highly conserved GAPGGG domain which acts as a effective immunogenic domain in humans [10]. Other features include a crucial Serine-177 phosphorylation site that is indispensible for HDV RNA replication and editing process [9]. An essential nuclear export signal (NES) region extends from amino acids 198-210 [11]. Within the NES, exists a clathrin box-binding domain from amino acids 199–203 which is responsible for clathrin-mediated endocytosis of HDV-RNA complex from nucleus to cytoplasm of infected hepatocytes. The amber/W-196 residue is indicated as the site for RNA editing that leads to formation of L-HDAg which inhibits HDV RNA replication and initiates viral assembly [7]. An isoprenylation site corresponding to C-terminal 19 amino acids of L-HDAg, undergoes farnesylation via Cystein-211, allowing export of L-HDAg to cytoplasm [9]. In the cytoplasm, the L-HDAg interacts with Hepatitis B surface antigen (HBsAg) in the form of clathrin-mediated vesicles at the endoplasmic reticulum, resulting in HDV virion maturation [11].

HDV genome is reported to undergo a higher range of genetic variability among all other RNA viruses. Previously, the genetic variability of HDV was based on the Restriction-Fragment Length Polymorphism (RFLP) of the R0 region of HDAg which assigned the viruses into genotypes I, II and III, while phylogenetic analysis allocates the HDV viruses to eight groups or clades (I to VIII) [5]. The heterogeneity in nucleotide sequence of the R0 region (delta antigen-coding region in the antigenomic strand of HDV RNA) is less than 14 to 15.7% among different isolates of the same genotypes, while between genotypes the heterogeneity ranges from 19 to 38%. About 30% divergence is observed in the amino acid sequences from different genotypes [12]. HDV genotype III (clade-3) is the most divergent group among HDV isolates [12,13].

Genotype I includes most of the European, North American, African and some Asian HDV isolates. Genotype II has been found in Japan and Taiwan, while genotype III has been found exclusively in South America. HDV genotype I-associated disease patterns are highly variable, ranging from severe to mild. Genotype II has been found only in East Asia and has been suggested to be associated with a milder disease course. Genotype III is particularly severe and results into fulminant hepatitis [2,14].

Hepatitis B and Hepatitis D dual infection is one of the serious viral health problem in Pakistan specifically in the province of Sindh where its incidence rate has reached up to about 67% [15]. The main cause of this spread is the unrestricted use of infected syringes and razor blades [14,16].

Even with such a high prevalence rate of HDV in this region, to the best of our knowledge, no phylogenetic-based study has been carried out on the predominant HDV isolates of Pakistan.

The aim of this study is to perform a preliminary analysis of the genetic relatedness of predominant Pakistani HDV isolates, to strains reported from different countries, assigned into the eight clades. The study will help greatly in identifying the variants present in our population and help in understanding the origin and spread of HDV infection in Sindh, Pakistan.

## Results

HDV isolates from patients diagnosed with chronic HDV were analyzed at the genetic level to determine the evolutionary relatedness of Pakistan HDV isolates to the reported strains of different countries and clades. This study was expected to identify new genetic variants that may exist in the local HDV strain.

### RFLP analysis

**Figure 1:**
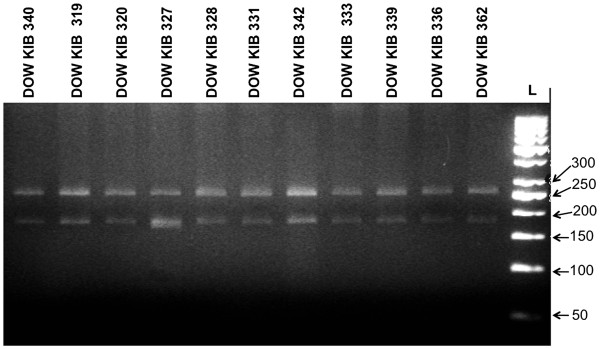
**HDV genotype characterization by RFLP of amplified R0 region for some strains of HDV isolated from serum of HDV patients.** The *Sma*I digest produced bands of 179 and 250 bp indicating genotype I. The *Sma*I site is present in the immunogenic region corresponding to nucleotides base pair 1066–1073 of HDAg.

Nested PCR amplified DNA digested with restriction enzyme *Sma*I produced two bands of 179 and 250 bp, demonstrating that all samples belonged to genotype I [5,17]. A representative sample of the digests is shown in (Figure 1).

### Phylogenetic analysis

**Figure 2:**
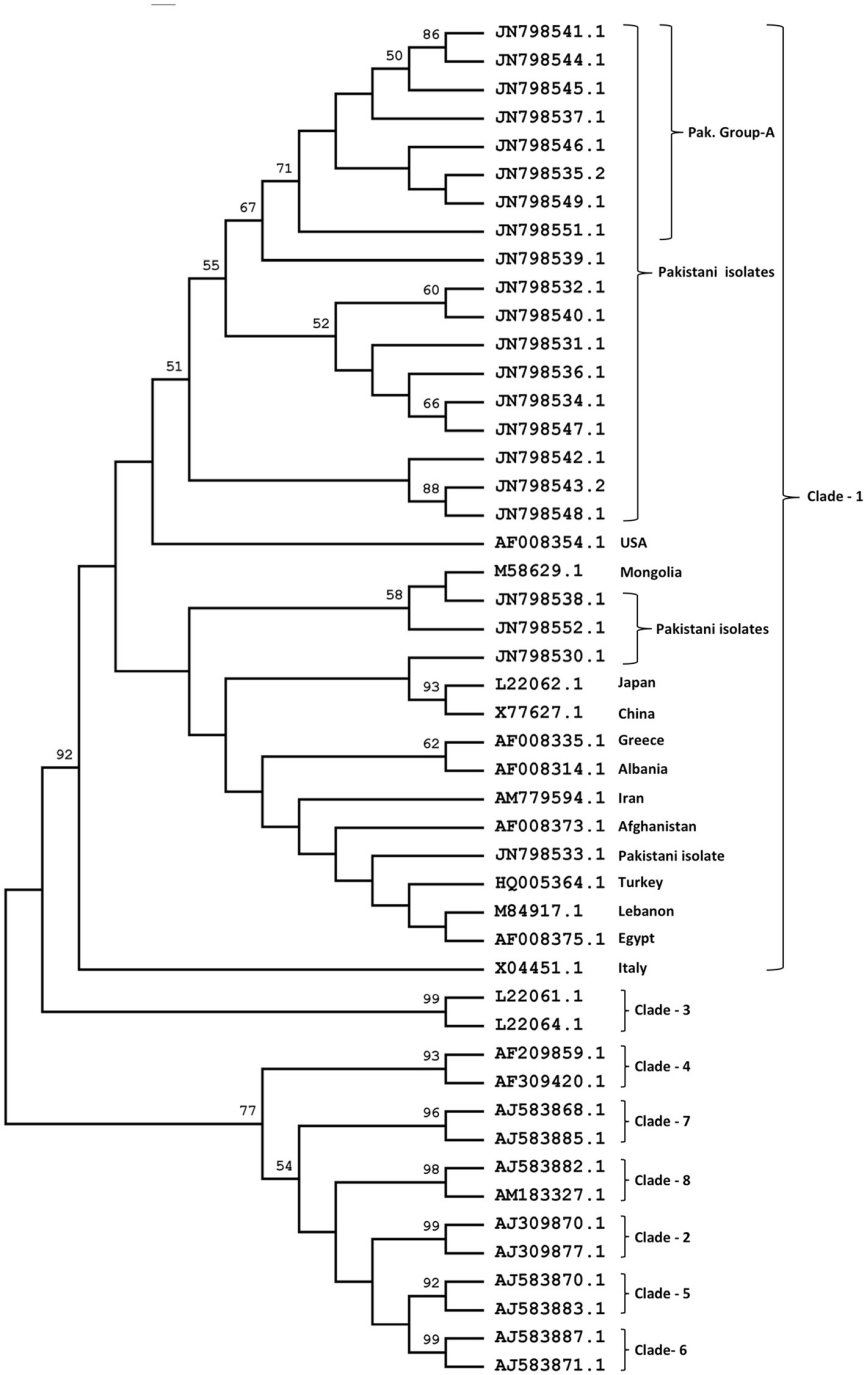
**Phylogenetic Analysis of L-HDAg nucleotide sequences from position 856–1282 of isolates from Karachi, Pakistan and reference sequences obtained from GenBank.** The analysis was done using the Maximum Likelihood method using the General Time Reversible gamma model, with bootstrap values (1000 replicates), shown next to the nodes of the tree. Evolutionary analyses were conducted in MEGA5.

**Figure 3:**
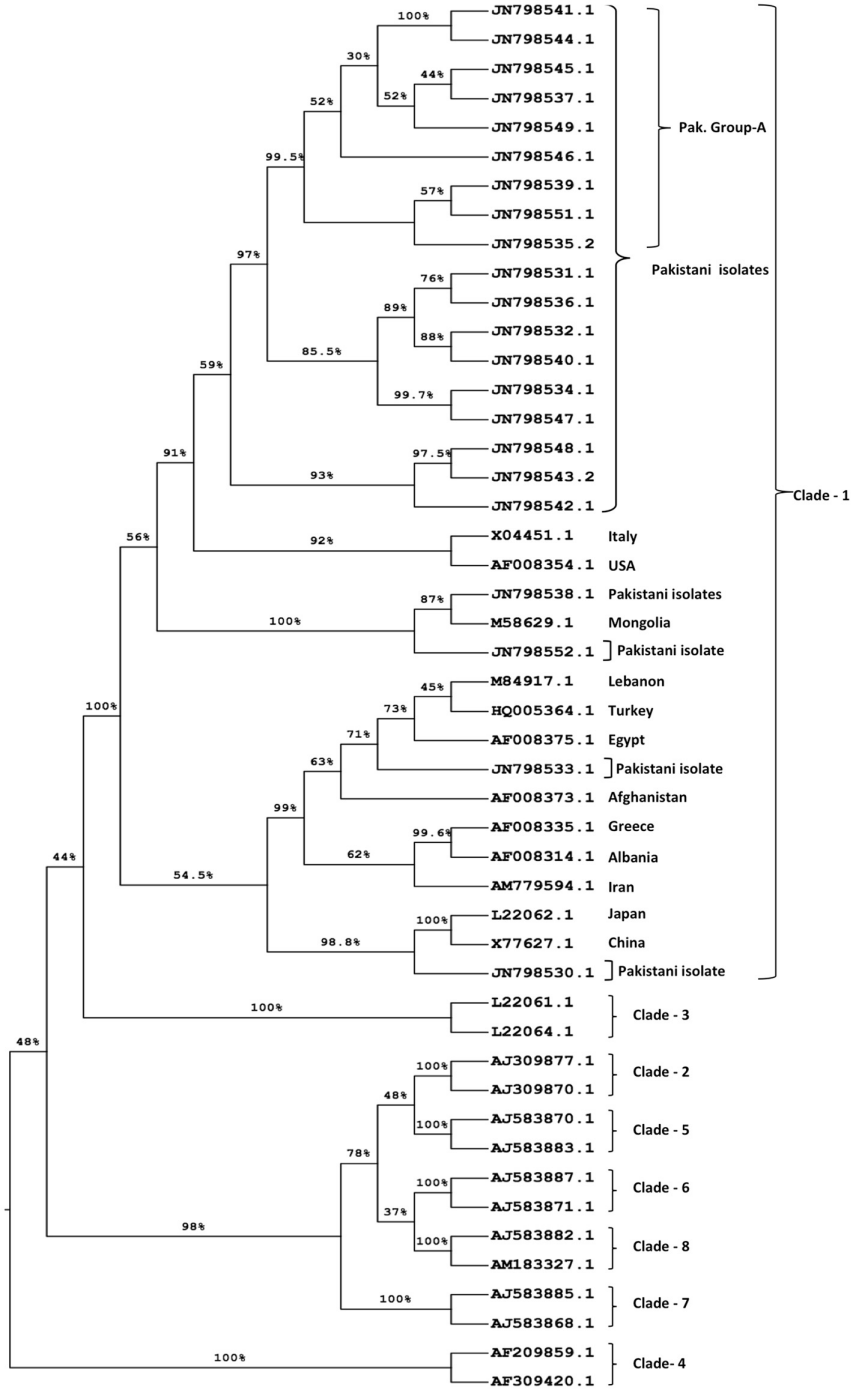
**Phylogenetic Analysis of L-HDAg nucleotide sequences from position 856–1282 of isolates from Karachi, Pakistan and reference sequences obtained from GenBank.** The analysis was carried out using Bayesian method by applying the Markov Chain Monte Carlo simulation using the software BEAST ver. 1.7.2. The values at the nodes of the tree show the posterior probabilities of the analysis.

The phylogenetic tree constructed by using the Maximum Likelihood method based on the GTR + G model is shown in Figure 2, and Bayesian method is shown in Figure 3.

The phylogenetic analysis of C-terminal part of L-HDAg of isolates from this study places all our isolates within the largest clade I of HDV with a significant bootstrap confidence value of 92% (ML) and posterior probability 100% (BEAST v1.7.2). This observation supports our PCR-RFLP results (Figure 1), where all isolates screened produced the DNA digest bands that are indicative of genotype I [5,17].

Further observations have revealed that isolates with accession number JN798541, JN798544, JN798545, JN798537, JN798546, JN798535, JN798549, JN798539 and JN798551 (designated as Pak. isolates group A) are closely clustered with a higher bootstrap value of 67% (ML) and posterior probability 99% (BEAST v1.7.2), indicating the existence of a potential subgroup within clade I. Other isolates are showing association with some geographically distant isolates: JN798538 and JN798552 are closer in the tree to an isolate from Mongolia (M58629), JN798530 lies close to sequences from China and Japan and sequence JN 798533 is in proximity to sequences from the Middle East (Figure 2 and Figure 3).

Majority of our isolates including the potential subgroup Pak. group A, seem closer to the isolate from US on both phylogenetic trees. Outside Pak. group A, most of our isolates are forming two clusters but with lower bootstrap values.

### Amino acid sequence analysis

**Figure 4:**
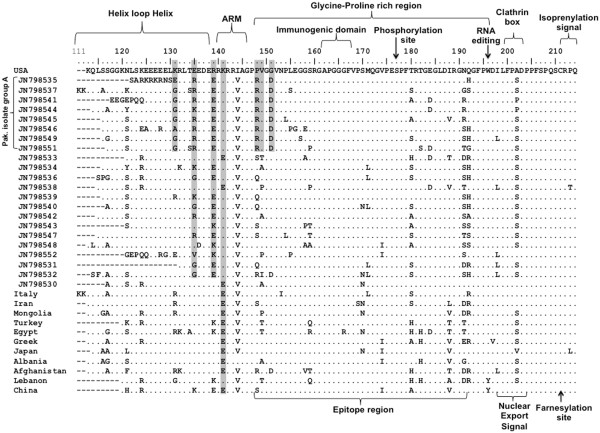
**Multiple Alignment of C-terminal region of L-HDAg predicted protein for Pakistani Isolates and other included reference strains of HDV-clade I.** Dots indicates conserved amino acids, Motifs related to functional properties are labeled, and atypical amino acids observed for L-HDAg of Pakistan Isolates are shown in shaded boxes.

The predicted amino acid sequence (111–214) of amplified region of our isolates corresponding to nucleotide 856–1282, were compared with reference sequences of Clade I, to check/confirm presence of all respective essential and conserved protein domains in this amplified region (Figure 4). All of the studied isolates express CRPQ region at C-terminal end of L-HDAg. CRPQ residues are specific for clade I and used to differentiate clade I from other clades of HDV [4,11]. This observation supports the phylogenetic outcome at the nucleotide level which had placed the Pakistani isolates into clade I.

It was also observed that the isolates which formed the subgroup within clade I on phylogenetic analysis specifically carry amino acid mutation/substitutions that are unusual to clade I (Figure 4).

## Discussion

The current study is the first phylogenetic analysis of HDV isolates from Pakistan. This evolutionary analysis places all of the studied isolates within the largest HDV clade I. Majority of the Pakistani isolates form a phyletic subgroup that branches away from most clade I isolates with a significant bootstrap and posterior probability value. This group is therefore more distant from most clade I sequences, but interestingly is more close to the clade I sequences from geographically distant areas (Italy and USA). Within this group, three clusters can be seen forming, one of which shows higher bootstrap and posterior probability values (shown as Pak. Group A) (Figures 2 and 3).

The region of interest in this study consists of several fundamental functional sites such as RNA binding domains (ARM region and HLH region), conserved motif (PESPF) with phosphorylation site at S-177, epitope protein, immunogenic region, clathrin box binding domain, RNA editing or Amber/W site, farnesylation site and isoprenylation site [2,8,9,11].

Multiple sequence alignment (MSA) of clade I predicted amino acid reference sequences and our sequences highlighted some well-conserved regions as well as some amino acid substitutions within critical regions of HDAg protein. Among these fundamental sites, the regions which are crucial for HDV survival including S-177, RNA editing or Amber/W site, immunogenic region and isoprenylation site were found to be highly conserved. However, most of our isolates showed variations in RNA binding domain composed of ARM and HLH region while isolates forming subgroup also show variation in the epitope (Glycine-Proline rich) region (Figure 4).

The MSA revealed that at positions 135 that lie within the HLH region and at position 139 at the start of the ARM region, very little or no variation was present in the reference sequences. At position 142 of the ARM region, amino acid K predominated in our strains while E was the main residue in the reference strains. Similarly, amino acid 148 at the beginning of the epitope region of HDAg was more varied (S, Q and R) in our strains whereas less variation was seen in the reference sequences (Figure 4).

The potential subgroup named Pak. Isolate group A, which became evident by the ML and Bayesian phylogentic analysis at the nucleotide level showed some unusual patterns of amino acid residues. Among the various amino acid substitutions, the group shows few atypical substitutions that stand out in the alignment. At amino acid 131, the G residue was predominant in this group while K residue was more frequent at this site in other strains. In the HDAg protein epitope region, the amino acid 148 in our sequence alignment showed that this group invariably had R residue, while P was the more frequent residue. Position 149 show residue V in the subgroup consistently, while greater degree of polymorphism was present in our other strains and the reference strains. In the same region, at position 151, residue D dominated in the subgroup while G was the main amino acid in all other strains (Figure 4). Such amino acid substitutions have not been reported for HDV in other studies.

Our study clearly shows that most of the isolates obtained from Karachi, Pakistan and its outskirts belong to clade I. This result is in accordance with previous study on HDV genotypic analysis of Pakistan [18]. The clustering of sequences of some of our isolates may point towards formation of a subgroup within clade I. Moreover, the amino acid substitutions discussed above lie in a critical region of HDAg involving RNA replication and packing. Such trends of amino acid substitutions in critical regions of the virus merit further investigation on a large scale supported by extensive clinical studies in order to ascertain their influence on the overall survivability and/or pathogenecity of the virus. Such studies can also determine if these substitutions are hallmark of the HDV strains of the region.

## Conclusions

Phylogenetic analysis of viral genomic sequences can be a forceful approach for the identification of transmission chains in order to control viral epidemics. In our studied isolates we observed a genetic diversity along with formation of potential subgroup within clade I. As viral hepatitis has taken an endemic form in the rural population of Sindh province of Pakistan, it is suggested that studies based on comprehensive demographic information and phylogenetic analysis may lead to provide further information about subgroup formation within clade I. Such studies can be an effective tool in checking and/or preventing the spread of hepatitis D virus infection.

## Methods

### Sample collection and storage

A study was designed and carried out to determine genotype variants of HDV isolates from the city of Karachi, Pakistan and its outskirts. Whole blood samples were obtained from 22 patients from the outpatient clinics of Dow University of Health Sciences (DUHS). These patients were diagnosed with chronic Hepatitis delta and included 16 males (mean age 32) and 6 females (mean age 22). The sampling was carried out under the approval of ethical committee of the institute. All the procedures were explained to the patients and/or their attendants and a written informed consent was obtained for this study. A copy of the written consent is available for review by the Editor-in-Chief of this journal.

About 5 cc of whole blood was collected from each subject in yellow geltop tubes. Serum was separated from whole blood by centrifugation of the geltop tubes at 1200 rpm for 10 minutes. After centrifugation serum was aliquoted and stored at −80°C. Related clinical parameters of patients regarding HBsAg, HBeAg and HCV status is presented in Table 1. HBAgs and HCV were evaluated by using an Enzyme –linked immunosorbent assay (ELISA kits, Diasorin S.p.A, Italy).

**Table 1:** Related parameters of hosts (patients) which were the source of the isolated HDV virus

**Host (patient) Sample ID.**	**GenBank Accession No**	**Age**	**Sex**	**HCV**	**HBsAg/HBeAg status**	ALT (IU/L)*
DOWKIB336	JN798530	50	M	No	+/−	117
DOWKIB359	JN798531	12	M	No	+/−	94
DOWKIB306	JN798532	36	M	No	+/−	88
DOWKIB262	JN798533	40	M	No	+/−	111
DOWKIB331	JN798534	45	M	No	+/−	78
DOWKIB300	JN798535	38	M	No	+/−	65
DOWKIB335	JN798536	18	M	No	+/−	106
DOWKIB319	JN798537	40	M	No	+/−	59
DOWKIB320	JN798538	24	F	No	+/−	127
DOWKIB318	JN798539	26	F	No	+/−	113
DOWKIB326	JN798540	35	F	No	+/−	101
DOWKIB342	JN798541	45	M	No	+/−	74
DOWKIB314	JN798542	18	F	No	+/−	134
DOWKIB323	JN798543	14	F	No	+/−	98
DOWKIB339	JN798544	24	M	No	+/−	NA
DOWKIB313	JN798545	45	M	No	+/−	72
DOWKIB327	JN798546	20	F	No	+/−	67
DOWKIB328	JN798547	18	M	No	+/−	127
DOWKIB357	JN798548	34	M	No	+/−	NA
DOEKIB 362	JN798549	21	M	No	+/−	63
DOWKIB309	JN798551	30	M	No	+/−	NA
DOWKIB333	JN798552	25	M	No	+/−	82
	* normal range 10 – 40 IU/L					

### RNA extraction and reverse transcriptase PCR

Viral RNA was extracted from 140 μl of serum by using QIAamp Viral RNA Mini Kit (Qiagen, Germany)**.** For cDNA synthesis by reverse transcription, 10 μl extracted RNA was mixed with 2 μl random primers (Fermentas) and denatured at 70°C for 10 minutes. To the denatured RNA/random primer, 8U AMV Reverse Transcriptase, (Fermentas), 25U RNAse inhibitor (Fermentas), 2.5 mM dNTPs (Fermentas) was added in a final reaction volume 20 μl. The reaction was then incubated at 42°C for 90 minutes [17,18].

### Primer designing

Pair of outer and inner primers containing both forward and reverse primers for first and second round was designed by using the web-based software Primer 3 [19]. (Table 2) represents the name, position, and sequences of designed primers.

**Table 2:** HDV Primer sequences used for Reverse-Transcription Nested PCR

**Primer Name**		**Primer Sequence; 5 - 3**	**Position on the HDV Genome**
Round-1	HDV-1 FOR	TCCCTTAGCCATCCGAGTGGAC	819 - 840
	HDV-1 REV	AGGGTTCACCGACAAGGAGAGG	1303 - 1285
Round-2	HDV-2 FOR	GGATGCCCAGGTCGGACCG	856 - 875
	HDV-2 REV	AAGGAAGGCCCTCGAGAACAAG	1282 - 1257

### RT-Nested PCR

One fourth of the synthesized cDNA was used to amplify the HDAg region extending from (856-1282 bp) by Nested PCR. The first round reaction mixture contained 2 μl each of 20 μM Round-1 forward and reverse primer, 0.5 μl (2.5U) of Taq DNA polymerase, (Fermentas), 2.5 μl of 10X PCR buffer and 1 μl of 10 mM dNTPs (Fermentas) in a total reaction volume of 25 μl. The reaction mix was cycled 94°C for 1 minutes (one cycle), 94°C for 1 minute, 55°C for 1 minutes, 72°C for 1 minute (35 cycles) and 72°C for 10 minutes (one cycle).

After completion of the first round of amplification, 4 μl of first round amplified PCR product was transferred to a new reaction tube for the second round of amplification. The second round nested fragment was amplified under the same conditions described for the first round of amplification [17,18].

Second round amplicons were resolved and screened using 2% agarose gel electrophoresis method. All PCR reactions were performed with appropriate negative controls to avoid any false positive results.

### PCR-RFLP genotyping

In order to analyze genetic variants of HDV strains, reverse transcriptase nested PCR amplified DNA product of second round was digested with the appropriate restriction enzyme. The reaction mix contained 5 μl of the amplified product in 1X restriction enzyme buffer with 2U *Sma*I (Fermentas) restriction enzyme, in a total volume 20 μl. The reaction mixture was then incubated overnight at 37°C and digested products were resolved by electrophoresis on a 3% agarose gel [17,18].

### DNA sequencing

All nested PCR fragments were purified by PCR cleanup system (Bioneer, Daejeon, Korea) and sent for commercial sequencing. Each PCR products were sequenced in both forward and reverse directions using the Round-2 forward and reverse PCR primers. Sequencing was carried out using the Genetic Analyzer ABI 3300, (Applied Biosystem), at the Centre for Applied Molecular biology CAMB, Lahore, Pakistan. Sequence data of twenty two samples was submitted to the NCBI GenBank and are available as accession numbers JN798530 to JN798549, JN798551 and JN798552.

### Nucleotide sequence analysis

Twenty six nucleotide sequences of HDAg coding (R0) region were obtained from GenBank to use as reference sequences, for phylogenetic analysis. The DNA sequences were chosen such that they represented the eight clades of HDV, with more sequences belonging to clade I. Twelve sequences belonged to clade I while fourteen belonged to clade II to clade VIII.

### Phylogenetic analysis

First, a multiple sequence alignment (MSA) was carried out for all C-terminal L-HDAg coding DNA sequences using ClustalW2 function of MEGA software version 5.0 (MEGA 5) [20]. The MSA included sequences from the new HDV isolates and reference sequences from GenBank. The default gap opening penalty (GOP) of 15 and gap extension penalty (GEP) of 6.66 were used. The MSA was then checked and edited manually for any inconsistency in the homologous alignment, using the alignment explorer window of MEGA 5. The program jModeltest version 0.1.1 [21] was used to determine the nucleotide substitution model and parameters that best fitted the dataset according to Akaike Information Criterion (AIC) and Bayesian Information Criterion (BIC). The general time reversible model with gamma distribution (GTR + G) was then used to create a maximum likelihood (ML) tree using MEGA 5.0, with bootstrap resampling (1000 replicates) in order to confirm reliability of phylogenetic tree. A Bayesian phylogenetic analyses (using GTR + G model) was also done by applying Markov Chain Monte Carlo simulation using the software BEAST v.1.7.2 [22]. The MCMC chain ran for 10 million states and sampled every 1000 states, with 10% burn-in. The results were examined in the software Tracer v1.5 [23], which showed an effective sample size (ESS) of >300 indicating a better convergence of all parameters. The maximum clade credibility (MCC) tree was obtained by using TreeAnnotator in the BEAST package [22]. Trees were visualized using the FigTree v1.3.1 program [24].

### Amino acid sequence analysis

For amino acid sequence analysis, the twenty two nucleotide sequences of the local isolates were translated by using ExPasy online tool [25]. The predicted amino acid sequences from the twelve clade I nucleotide sequences used in the phylogenetic analysis were obtained from GenBank. Multiple sequence alignments of the 34 amino acid sequences were performed using ClustalW2 function of MEGA 5, in order to delineate the consensus sequences. The predicted protein sequence of the nucleotide sequence AF008354.1 (shown as USA in Figure 4), was chosen as a base reference for the amino acid MSA due to its closeness with most of our isolates in the nucleotide phylogenetic tree.

## Competing interests

The authors declare that they have no competing interests.

## Authors’ contributions

SP searched the literature, performed the benchwork and drafted the manuscript, IN helped in the sample collection, provided the optimized protocols and methods, SMS participated in sample collection and analysis of biochemical parameters of patients. AA analyzed the data and helped in paper write up. OYK designed the study and critically viewed the manuscript. All authors have read and approved the final manuscript.

## References

[B1] Hepatitis D[http://www.who.int/csr/disease/hepatitis/whocdscsrncs20011/en/index5.html]

[B2] Farci P Delta hepatitis: an update J Hepatol 2003 39 Suppl 1 S212 219 14708706 10.1016/s0168-8278(03)00331-3

[B3] Hepatitis D Virus[http://www.stanford.edu/group/virus/delta/2005/]

[B4] Huang CR Lo SJ Evolution and diversity of the human hepatitis D virus genome Advances in Bioinformatics 2009 2010 1 9 10.1155/2010/323654 PMC2829689 20204073

[B5] Deny P Hepatitis delta virus genetic variability: from genotypes I, II, III to eight major clades? Curr Top Microbiol Immunol 2006 307 151 171 10.1007/3-540-29802-9_8 16903225

[B6] Pascarella S Negro F Hepatitis D virus: an update Liver International 2011 31 7 21 10.1111/j.1478-3231.2010.02320.x 20880077

[B7] Casey JL RNA editing in hepatitis delta virus Curr Top Microbiol Immunol 2006 307 67 89 10.1007/3-540-29802-9_4 16903221

[B8] Shakil AO Hadziyannis S Hoofnagle JH Di Bisceglie AM Gerin JL Casey JL Geographic distribution and genetic variability of hepatitis delta virus genotype I Virology 1997 234 160 167 10.1006/viro.1997.8644 9234957

[B9] Lai MM Handa H, Yamaguchi Y Hepatitis Delta Antigen: Biochemical Properties and Functional Roles in HDV Replication Hepatitis Delta Virus 2006 Springer 38 51

[B10] Wang JG Jansen RW Brown EA Lemon SM Immunogenic domains of hepatitis delta virus antigen: peptide mapping of epitopes recognized by human and woodchuck antibodies J Virol 1990 64 1108 1116 1689390 10.1128/jvi.64.3.1108-1116.1990 PMC249224

[B11] Greco-Stewart V Pelchat M Interaction of Host Cellular Proteins with Components of the Hepatitis Delta Virus Viruses 2010 2 189 212 10.3390/v2010189 21994607 PMC3185554

[B12] Radjef N Gordien E Ivaniushina V Gault E Anais P Drugan T Trinchet JC Roulot D Tamby M Milinkovitch MC Deny P Molecular phylogenetic analyses indicate a wide and ancient radiation of African hepatitis delta virus, suggesting a deltavirus genus of at least seven major clades J Virol 2004 78 2537 2544 10.1128/JVI.78.5.2537-2544.2004 14963156 PMC369207

[B13] Casey JL Gerin JL Genotype-specific complementation of hepatitis delta virus RNA replication by hepatitis delta antigen J Virol 1998 72 2806 2814 9525600 10.1128/jvi.72.4.2806-2814.1998 PMC109725

[B14] Abbas Z Jafri W Raza S Hepatitis D: Scenario in the Asia-Pacific region World J Gastroenterol 2010 16 554 562 10.3748/wjg.v16.i5.554 20128022 PMC2816266

[B15] Khan AU Waqar M Akram M Zaib M Wasim M Ahmad S Niaz Z Ali S Ali H Idrees M Bajwa MA True prevalence of twin HDV-HBV infection in Pakistan: a molecular approach Virol J 2011 8 420 10.1186/1743-422X-8-420 21888671 PMC3179753

[B16] Bosan A Qureshi H Bile KM Ahmad I Hafiz R A review of hepatitis viral infections in Pakistan J Pak Med Assoc 2011 60 1045 1058 21381562

[B17] Cakaloglu Y Akyuz F Bozaci M Ibrisim D Pinarbasi B Demir K Kaymakoglu S Besisik F Badur S Okten A Prevalence and clinical significance of SEN-H virus in chronic hepatitis B, C and delta infections in Turkey Turk J Gastroenterol 2008 19 104 108 19110665

[B18] Moatter T Abbas Z Shabir S Jafri W Clinical presentation and genotype of hepatitis delta in Karachi World J Gastroenterol 2007 13 2604 2607 17552010 10.3748/wjg.v13.i18.2604 PMC4146823

[B19] Primer 3 [http://primer3.sourceforge.net/history.php]

[B20] Tamura K Peterson D Peterson N Stecher G Nei M Kumar S MEGA5: molecular evolutionary genetics analysis using maximum likelihood, evolutionary distance, and maximum parsimony methods Mol Biol Evol 2011 28 2731 2739 10.1093/molbev/msr121 21546353 PMC3203626

[B21] D P jModelTest.Phylogenetic Model Averaging Molecular Biology and Evolution 2008 25 1253 1256 10.1093/molbev/msn083 18397919

[B22] Drummond AJ Rambaut A BEAST: Bayesian evolutionary analysis by sampling trees BMC Evol Biol 2007 7 214 10.1186/1471-2148-7-214 17996036 PMC2247476

[B23] Rambaut A Drummond AJ Tracer v1.5 2009 Available from http://beast.bio.ed.ac.uk/Tracer. Accessed 24 July 2012

[B24] Rambaut A FigTree v1.3.1: Tree figure drawing tool 2008 Available: http://tree.bio.ed.ac.uk/software/figtre?e/. Accessed 24 July 2012

[B25] ExPASy - Translate tool[web.expasy.org/translate/]

